# Never Too Old for Congenital Heart Disease: A Case of Cor Triatriatum Sinistrum and Mitral Valve Prolapse

**DOI:** 10.7759/cureus.21898

**Published:** 2022-02-04

**Authors:** Mariana Saraiva, Ana Rita Moura, M Luz Pitta, Vitor Martins

**Affiliations:** 1 Cardiology, Hospital Distrital de Santarém, Santarém, PRT

**Keywords:** 3d imaging, adult congenital heart disease (achd), multimodality cardiac imaging, cor triatriatum sinister, mitral valve prolapse

## Abstract

Cor triatriatum is a congenital atrial abnormality, which comprises a membrane that divides the atrium into two chambers. Symptoms can arise either due to the obstructive nature of this membrane or related to other associated anomalies, such as atrial septal defects or abnormal pulmonary venous return.

The authors report the case of an adult male, in whom an incidental finding of cor triatriatum sinistrum was documented in association with mitral valve prolapse. However, both the late diagnosis and the association with mitral valve disease are uncommon.

Multimodality imaging evaluation can prove very helpful in these cases to better define the anatomy of the left atrium and appropriately plan for intervention when indicated.

## Introduction

Cor triatriatum is a congenital abnormality where a septum divides the atrium into two cavities, giving rise to a heart with three atrial chambers. This rare anomaly (0.1% of all diagnosed congenital abnormalities) can be found either on the left (sinistrum) or the right (dextrum) atrium, but cor triatriatum sinistrum is more frequent [1].

Most cases are diagnosed during childhood, mainly when this membrane causes obstruction to the blood flow, from the upper chamber, where the venous drainage takes place, to the lower chamber, in relation with the atrioventricular valve. The presence of symptoms is also related to other associated congenital abnormalities, such as atrial septal defects, abnormal pulmonary or systemic venous return, or more complex lesions, with single ventricle physiology [2]. Surgical treatment is curative and indicated when symptomatic.

The authors present the case of an adult with mitral valve prolapse and an incidental finding of cor triatriatum sinistrum.

## Case presentation

A 67-year-old male with a history of uncontrolled dyslipidemia and a former smoker was referred to Cardiology after detecting an irregular pulse and a heart murmur. He had no relevant family history and complained only of infrequent palpitations. However, upon examination, a holosystolic murmur was audible in the apex. Also, an arrhythmic pulse was present in relation to frequent ventricular ectopic beats, evident on ECG, with no other relevant changes. 

**Figure 1:**
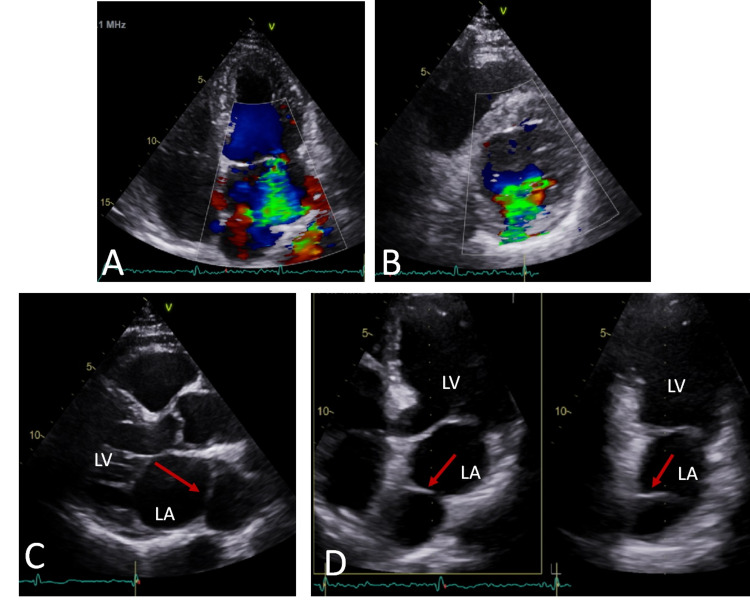
Transthoracic ECG showing both the presence of severe mitral regurgitation (top row) and cor triatriatum sinistrum (lower row; red arrow). A) Apical 4-chamber view; B) Parasternal short-axis view; C) Parasternal long-axis view; and D) Three-dimensional biplanar apical view. LA: Left atrium; LV: Left ventricle.

The transthoracic ECG showed a severe mitral regurgitation, in the context of an anterior mitral leaflet prolapse, as well as bi-atrial dilation, mild left ventricle dilation (biplane volume 78 mL/m2, end-systolic diameter 42 mm), with preserved ejection fraction (65% Simpson biplane) and low pulmonary hypertension probability. Also, a membrane was noted in the left atrium, dividing this chamber into two, suggestive of cor triatriatum sinistrum (Figure 1). 

Holter monitoring revealed very frequent ventricular ectopic beats (12336) and two short runs of non-sustained monomorphic ventricular tachycardia, with symptomatic improvement for which anti-arrhythmic medication was prescribed.

Routine blood workup did not find relevant changes, aside from elevated N-terminal prohormone of brain natriuretic peptide (NTproBNP) levels (2246 ng/mL, normal range <125 ng/mL).

**Figure 2:**
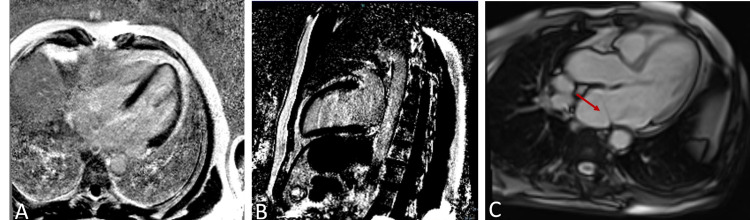
Cardiac magnetic resonance showing no myocardial fibrosis and confirming the presence of cor triatriatum sinistrum (red arrow). No late gadolinium enhancement in both 4-chamber (A) and 2-chamber views (B); 5 chamber T1-imaging (C), showing a membrane dividing the left atrium.

Given the presence of cardiovascular risk factors and significant ventricular arrhythmia, a cardiac magnetic resonance with perfusion imaging was performed. It did not show inducible ischemia or late gadolinium enhancement and confirmed the transthoracic echocardiography's previous findings (Figure 2).

**Figure 3:**
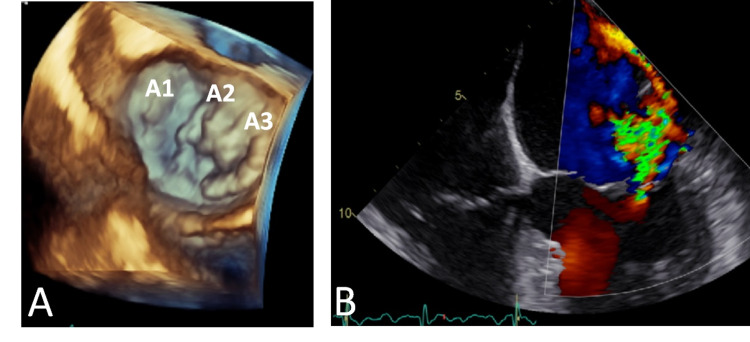
Transesophageal echocardiography showing a severe mitral regurgitation, due to anterior leaflet prolapse at A2 and A3 scallops. The eccentric jet, directed laterally and posteriorly, reaches the left atrium's ceiling. A) Three-dimensional imaging of the mitral valve, atrial view; B) mid-esophageal 4-chamber view (0º) with colour Doppler.

**Figure 4:**
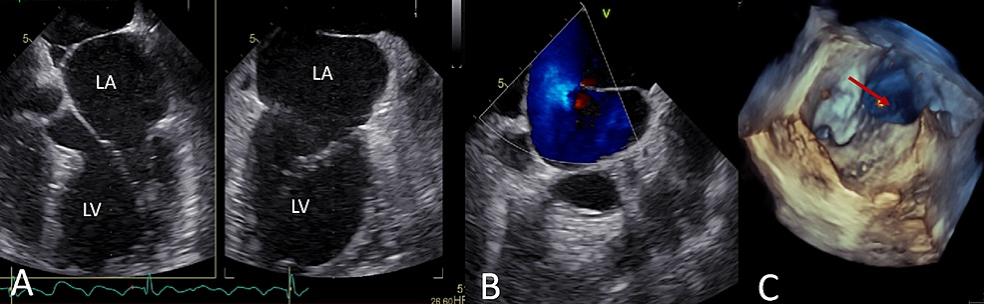
Transesophageal echocardiography characterizing the cor triatriatum sinistrum. The LA is partially divided by a nonrestrictive membrane, shaped like a diaphragm, with a single posterior wide opening (red arrow). A) Biplanar mid-esophageal view; B) modified mid-esophageal view at 90º; C) Three-dimensional reconstruction of the septum, atrial view. LA: Left atrium; LV: Left ventricle.

The patient was referred for transesophageal echocardiography to further characterize the mitral valve anatomy. The mitral valve prolapse, involving A2 and A3 scallops, accounted for the eccentric mitral regurgitation (Figure 3). Also, an incomplete nonobstructive septum was noted in the upper anterior portion of the left atrium (Figure 4); other possible associated abnormalities were excluded.

He was diagnosed with atrial flutter and started on oral anticoagulation during follow-up. However, he remained asymptomatic, with good functional capacity on exercise testing. Valve intervention was discussed, and a conservative approach with close follow-up was decided.

## Discussion

Triatrial heart or cor triatriatum is the name of the condition where septation divides one of the atria into two chambers, thus forming a heart with three atria.

In the case of the cor triatriatum sinistrum, the upper chamber contains the pulmonary veins, and the lower chamber connects with the left atrial appendage and the mitral valve. The embryological explanation for the presence of the septum is not yet fully understood. However, the most widely accepted explanation is the "malincorporation" theory, which advocates that there is abnormal incorporation of the common pulmonary vein in the left atrium [1]. No risk factors or genetic syndrome associations have been described.

The septum is usually a fibro-muscular membrane that can assume different shapes (diaphragmatic, hourglass, or tubular) and can either be intact or have one opening or multiple fenestrations. The proposed classifications, such as Loeffler's and Lucas' classification, take into account the anatomy and physiology of the membrane, the communication with the lower chamber, the pulmonary venous drainage, and the presence of other associated abnormalities [3,4].

These same aspects also determine the presence of symptoms, mainly when the membrane itself has a small, restrictive opening or other associated congenital lesions. In the first case, cor triatriatum sinistrum mimics the presentation of mitral stenosis, with dyspnea, orthopnea, or hemoptysis and accounting for evidence of pulmonary congestion, but in the absence of an opening snap or a loud S1. The clinical presentation can be particularly severe in children with recurrent respiratory distress, wheezing, and failure to thrive [5]. Other symptoms can appear according to the physiology of the associated congenital abnormalities.

As previously mentioned, the diagnosis is rare in adults, but the isolated form or classical type, with a single wide opening, is the most frequent in this age group. Nevertheless, with progressive thickening and calcification, it can become clinically relevant. Also, atrial arrhythmias and embolic events can appear due to the enlargement of the atrium, mainly in relation to atrial fibrillation [1].

Echocardiography, particularly with three-dimensional imaging, plays a significant role in diagnosis since it can provide comprehensive anatomical evaluation and functional assessment, and screening of associated abnormalities. In addition, cardiac CT and MRI can offer relevant additional information, mainly when there are multiple defects or a history of previous interventions, with cardiac catheterization being of little additional value nowadays [6]. 

Surgical correction is advised in the presence of symptoms, either related to the defect itself or the associated abnormalities. In a few selected cases, with favorable anatomy, balloon dilation can be considered [7].

Our case illustrates the classical type, where cor triatriatum sinistrum was an incidental, late diagnosis associated with mitral valve disease. However, given that the patient had no functional limitation, with preserved ejection fraction and only mild left ventricle dilation, a decision for clinical surveillance was made. Nevertheless, the diagnosis of cor triatriatum sinistrum may prove relevant since it will ensure better surgical planning when a mitral valve repair is advised. 

Multimodality imaging evaluation is key in complex cases of congenital heart disease. In our case, this strategy proved helpful and warranted full characterization of both the congenital heart defect and valve disease.

## Conclusions

Cor triatriatum sinistrum is a rare congenital abnormality uncommon in adults. The presentation in adulthood depends on anatomical and functional aspects and associated congenital abnormalities. Although also infrequent, its association with mitral valve disease can occur.

Multimodality imaging should be the preferred strategy to assess complex congenital heart disease cases, mainly when intervention is considered.​​
